# Exploring the Potential of Concept Associations for the Creative Generation of Linguistic Artifacts: A Case Study With Riddles and Rhetorical Figures

**DOI:** 10.3389/fpsyg.2018.01792

**Published:** 2018-09-26

**Authors:** Virginia Francisco, Raquel Hervás, Gonzalo Méndez, Paloma Galván

**Affiliations:** ^1^Departamento de Ingeniería del Software e Inteligencia Artificial, Universidad Complutense de Madrid, Madrid, Spain; ^2^Instituto de Tecnología del Conocimiento, Universidad Complutense de Madrid, Madrid, Spain

**Keywords:** concept associations, computational creativity, rhetorical figures, riddles, metaphor, simile, analogy

## Abstract

Automatic generation of linguistic artifacts is a problem that has been sporadically tackled over the years. The main goal of this paper is to explore how concept associations can be useful from a computational creativity point of view to generate some of these artifacts. We present an approach where finding associations between concepts that would not usually be considered as related (for example *life* and *politics* or *diamond* and *concrete*) could be the seed for the generation of creative and surprising linguistic artifacts such as rhetorical figures (*life is like politics*) and riddles (*what is as hard as concrete?*). Human volunteers evaluated the quality and appropriateness of the generated figures and riddles, and the results show that the concept associations obtained are useful for producing these kinds of creative artifacts.

## 1. Introduction

One of the most remarkable passages in *The Hobbit* (Tolkien, 1937) takes place in the riddle competition between Bilbo and Gollum, with one of the trickiest riddles being:

*Voiceless it cries*,
*Wingless flutters*,
*Toothless bites*,
*Mouthless mutters*.

It is noteworthy to see how each verse transfers some properties from other entities to the solution of the riddle - the wind - in such a way that it makes it look amazingly enigmatic.

The possibility to transfer similar properties between concepts from different domains is a literary resource that has been exploited by writers all over the years, even before Aristotle defined metaphors as “*giving the thing a name that belongs to something else; the transference being either from genus to species, or from species to genus, or from species to species, or on grounds of analogy.”* However, computational approaches to riddle or rhetorical figure generation have not explicitly made extensive use of this mechanism in order to create literary artifacts. Concept relations have proved to be useful for rhetorical figure analysis but have not been explored in depth for rhetorical figure generation, for example.

With the increasing growth of the Computational Creativity research field, and the development of new knowledge bases with linguistic resources coming from the area of computational linguistics, new approaches are arising that are shedding more light to solve the problem of how rhetorical figures and riddles can be automatically created and that, at the same time, are as meaningful for the human mind as those created by humans. In knowledge bases where concepts have associated properties, there is a large amount of comparative information that is implicitly encoded in the values of the properties that these concepts share. This kind of information can be useful in tasks where it is required to transfer similar properties between concepts from different domains, such as the generation of rhetorical figures (e.g. *this shirt is as white as snow*) or the generation of riddles based on comparisons (e.g. *What is as big as an elephant and weighs as little as smoke?*).

This paper presents a new approach to explore the use of concept associations from a computational creativity point of view. The idea is that finding associations between concepts that would seem unrelated could be the seed for the generation of creative artifacts. This idea is implemented by exploring the properties and categories of an initial concept, and finding other concepts that share some of these properties with different saliency. This study addresses the potential of this approach for the automatic generation of two kinds of creative artifacts: rhetorical figures and riddles. For example, we are able to create the analogy “*snow is as soft as a carpet”* for the target concept *snow* or the riddle “*What is as hard as concrete and as transparent as glass?”* for *diamond*.

The rest of the paper is organized as follows: section 2 presents work related to the topics addressed in this article. Section 3 describes our approach to finding concept associations and how it has been applied to the generation of riddles and rhetorical figures. The generated artifacts were evaluated as it is explained in section 4, and some conclusions and future work are drawn in section 5.

## 2. Related work

The work presented in this paper draws from the idea that concept associations based on similarity can be useful to generate linguistic artifacts, such as riddles and rhetorical figures. We have surveyed the areas of conceptual similarity, riddle generation and rhetorical figures generation and the most relevant works in these areas are described in the following subsections.

### 2.1. Conceptual similarity and its relation to creativity

Concepts share structures and properties which make them ideal candidates for creative operations such as analogy generation, conceptual blending or design.

Analogy is a cognitive process that transfers information or meaning from one concept (the source) to another concept (the target), or a linguistic expression corresponding to such process. Analogy is based on the mapping of the properties of source and target. This mapping takes place not only between objects, but also between relations of objects. Analogy plays a significant role in problem solving, decision making, generalization, creativity, invention and prediction, and lies behind basic tasks such as the identification of places, objects and people. It has been argued that analogy is “the core of cognition” (Gentner et al., 2001). Specific analogical language comprises comparisons, metaphors and similes. The Contemporary Theory of Metaphor (Lakoff, 1993) suggests that commonly used metaphorical expressions are surface realizations of an underlying conceptual metaphor and are understood via a cross-domain conceptual mapping between two concepts. The Structure Mapping Model (Gentner and Wolff, 1997) proposes that metaphors act to set up correspondences between conceptual structures of the concepts involved. More recently, Feldman (2008) has ellaborated the Neural Theory of Language. This theory treats language as a biological human ability and suggests ways in which language and thought may be realized in the brain, putting forward that many basic conceptual metaphors arise from embodied experiences, even before we learn to speak, as they map concepts in our brain rather than words in a sentence (Lakoff, 2012).

Conceptual blending (Fauconnier and Turner, 2003) is a basic mental operation that leads to new meaning. It plays a fundamental role in the construction of meaning in everyday life, arts and sciences. The same idea of mapping between source and target used in analogy is used by conceptual blending. The essence of conceptual blending (Fauconnier and Turner, 1998) is to match the mental spaces of two concepts and project them to a separate blended mental space, giving rise to a new concept. Mental spaces are small conceptual packets constructed as we think and talk, for purposes of local understanding and action.

The role of concept associations has also been studied in design. In order to generate a product, designers must select a source, discern properties of this source, and transfer this property to the product they are designing. The selection of a source is affected by the extent to which it represents the meaning the designer intends to convey (its salience) and the strength of its association with the product (their relatedness).

Therefore, if we intend to emulate creative operations such as those mentioned above, it is vital to have a way of mapping concepts and finding similarity between them. The structured resource WordNet (Miller, 1995) has a taxonomic organization of nouns and verbs, in which very general categories are successively divided into sub-categories. This structure allows us to measure the mapping information of two lexical concepts. Therefore, we can identify the deepest point in the taxonomy at which this content starts to diverge, which is called the Least Common Subsumer (LCS) of two concepts (Pedersen et al., 2004). Leacock et al. (1998) use the length of the shortest path between two concepts as a proxy for the conceptual distance between them. To connect two ideas in a hierarchical system, one must vertically ascend the hierarchy from one concept, change direction at a potential LCS, and then descend the hierarchy to reach the second concept. This way of understanding conceptual similarity is called vertical thinking (De Bono, 1970).

Vertical thinking reduces the similarity of two concepts to a single number which is poorly suited to creative comparisons (Veale and Li, 2013). When creativity comes into play as when we look for similarities between concepts to create rhetorical figures, the obvious similarities are not enough, it is necessary to look for new ways of seeing a concept, to find other not so obvious similarities. For example, if we look for the most similar concepts to *lawyer* we would get concepts like *defender* or *judge*, but if we intend to obtain *shark* as a *lawyer*-like concept we need to go further. To find novel and non-obvious similar concepts, one must use what De Bono (1970) calls lateral thinking. De Bono states that the best solution for creativity purposes is the combination of lateral and vertical thinking. Lateral thinking can be used to create a group of similar concepts from which one will then be selected by vertical thinking.

Thesaurus Rex^1^ is a system for the exploration of concepts that returns lateral views of a concept which are obtained from the web (Veale and Li, 2013). For example, to highlight the potential toxicity of *coffee*, Thesaurus Rex suggests, as concepts similar to coffee, *alcohol, tobacco* or *pesticide* because they are all categorized as toxic substances on the web.

### 2.2. Generation of rhetorical figures

Metaphors play an important role in communication, occurring as often as every third sentence (Shutova et al., 2012). However, although metaphors have been widely studied in Natural Language Analysis, this has not been the case in Natural Language Generation. There is a lot of work related to metaphor detection (Wilks et al., 2013), identification (Shutova et al., 2010), meaning (Glucksberg and McGlone, 2001; Vega Moreno, 2004; Terai and Nakagawa, 2008; Xiao et al., 2016), extraction and annotation (Wallington et al., 2003) but few related to metaphor generation. The reason can be that metaphor generation is as challenging as human creativity will allow.

In the field of Natural Language Generation, there have been a number of attempts to establish procedures for constructing rhetorical figures as important ingredients of generated spans of text. This has been attempted both in general terms (Hervás et al., 2006b) for different types of rhetorical figures, and for specific cases like analogies (Hervás et al., 2006a) or metaphors (Hervás et al., 2007). The attempts considered the problem of using rhetorical figures during text generation in general theoretical terms but lacked sufficient volume of explicit knowledge on the underlying semantics of words to be capable of practical generation.

An interesting feature of rhetorical figures is that they usually work independently of language. For example, the metaphor “*an argument is a war”* is used in different languages to express that arguments are as wars to be won. This has led to the hypothesis that the mapping between conceptual domains corresponds to neural mappings in the brain (Feldman and Narayanan, 2004; du Castel, 2015). This hypothesis, together with the recent development of sources of knowledge that allow easy mining of large corpora of text for significant word associations, has lead to the emergence of a number of systems that rely on these for constructing rhetorical figures of different types.

Jigsaw Bard (Veale and Hao, 2011) is a web service that exploits linguistic idioms to generate similes on demand. For any given property (or blend of properties), Jigsaw Bard presents a range of similes. To get these similes it scans Google n-grams to index potential idioms which are then re-purposed as a simile. For example, for the property *wet* Jigsaw Bard returns the idiom “*a lake of tears,”* that can be used to create simile like “*wet like a lake of tears,”* a melancholic way to accentuate the property *wet*.

Metaphor Magnet (Veale and Li, 2012) is a web service that allows users to enter queries such as “*Life is a +mystery,” “Google is -Microsoft”* or “*Steve Jobs is Tony Stark”*^2^. Each of the concepts of the query is expanded using the set of stereotypes that are commonly used to describe it. Then, its properties and those of its stereotypes are associated to the concept. The properties highlighted in the resulting metaphor are those that are at the intersection of the two concepts' properties. For example, in the case of “*Life is a +mystery,”* the properties *entertaining, thrilling, intriguing*, and *alluring* are all highlighted as being in the intersection of *life* and *mystery*.

Metaphor Eyes (Veale, 2014a) metaphorizes one concept as another concept. Given *scientist* and *explorer* it generates metaphors such as “*scientists make discoveries like explorers.”* It employs a propositional model of the world that reasons with subject-relation-object triples rather than subject-attribute pairs (as Metaphor Magnet does). Metaphor Eyes acquires its world-model from a variety of sources and it views metaphor as a representational lever, allowing it to fill the holes in its weak understanding of one concept by importing relevant knowledge from a neighboring concept.

Figure 8 (Harmon, 2015) is a system that contains an underlying model for what defines creative and figurative comparisons, and evaluates its own output based on these rules. The system is provided with a model of the current world and an entity in the world to be described. A suitable noun is selected from the knowledge base, and the comparison between the two nouns is clarified by obtaining an understanding via corpora search of what these nouns can do and how they can be described. Sentence completion occurs by intelligent adaptation of a case library of valid grammar constructions. Finally, the comparison is ranked by the system based on semantic, prosodic, and knowledge-based qualities. Figure 8 simulates the human-authoring process of revision by generating many figure variations for a single concept, and choosing the best among them.

### 2.3. Generation of riddles

Although the generation of riddles may seem a difficult task from a computational point of view, there are several attempts to the automatic generation of riddles that are presented in this section.

De Palma and Weiner (1992) propose a model of a knowledge representation that contains the data to generate or solve riddles. Its knowledge-base contains Concepts and RoleSets. The Concept is the primary representational entity. For example, MOUTH, RIVER-MOUTH, or PERSON-MOUTH. Concepts are connected to one another by links which indicates that the subordinate Concept (subConcept) stands in an inheritance and subsumption relationship with the superordinate Concept (superConcept). For example, PERSON-MOUTH is an ANIMAL-MOUTH and a MOUTH. RoleSets represent predicates of a Concept. For example, PERSON-MOUTH has the RoleSet EAT, meaning that a function of a person's mouth is to eat. They developed an algorithm that generated a riddle based on homophonous concepts in the following way: first, two homophonous concepts are searched in the knowdedge-base (for example, PERSON and RIVER which shared the fact that both have a MOUTH). Secondly, they look for a property that is not shared by both concepts (in the case of PERSON and RIVER we have that PERSON-MOUTH has EAT as a property but RIVER-MOUTH does not have this property). The result is a riddle of this type: “What has a mouth and cannot eat?”

JAPE (Joke Analysis and Production Engine) (Binsted and Ritchie, 1997; Ritchie, 2003) is a question-answer riddle generation system. To create riddles, JAPE uses templates with slots where words or phrases are inserted. To determine which words must be incorporated to the final riddle, the system makes use of predefined schemas (manually built from previously known jokes), which establish relationships words must hold to build a joke. The program was tested by 120 children that rated generated riddles, human-generated texts, and non-joke texts for “jokiness” and “funniness.” The evaluation confirmed that riddles generated were jokes, and that there is no significant difference in “funniness” or “jokiness” between punning riddles generated by their system and published human-generated jokes.

Some of the authors of JAPE have also developed STANDUP (Waller et al., 2009), a large-scale pun generator to allow children with communication disabilities to improve their linguistic skills. The pun generation followed the same steps used in JAPE, but several improvements had to be introduced in order to adapt the generated puns to the target audience, i.e. children with communication disabilities: speech output, picture support, restricted topics or use of familiar words, etc. The system was evaluated with real users over a short period, and although no positive effects could be observed on the long term, the authors report a change in the attitude of the children toward communication.

Guerrero et al. (2015) present a Twitter bot that generates riddles about celebrities. The model selects a celebrity, retrieves relevant attributes to describe her, generates analogies between her attributes and converts such descriptions into utterances, and, finally, tweets the generated riddle and interact with users by evaluating their answers. The attributes of the celebrities are retrieved from well-structured sources, such as the Non-Official Characterization (NOC) list (Veale, 2015), and from poorly-structured sources, such as Wikipedia. All the attributes obtained are filtered, only a subset of unique and interesting attributes are considered. A subset of features is considered unique if they describe only one celebrity and is considered interesting when it describes a character with attributes that altogether represent relevant traits, but do not provide excessive information so that the riddle cannot be easily guessed. To evaluate the riddle generation they asked 86 people to evaluate five riddles. They first asked the participants to guess the answer to the riddle. Then, they presented the correct answer and asked if they knew the person in question. The participants indicated whether they considered the quality of the riddle satisfactory and, if negative, gave the reason why it was not good. The percentage of known celebrities once the answer was presented (54.19%) indicates that the process for the selection of celebrities should be improved. The low number of correct answers (15.58%) suggests that the complexity of the generated riddles was high.

## 3. Finding concept associations for creative purposes

As seen in the previous section, mapping concepts is the basis of some creative operations such as rhetorical figures generation, conceptual blending or design. Taking this into account, the main purpose of this work is the exploration of how concept associations can be useful from a computational creativity point of view. The underlying idea is that finding associations between concepts that are initially considered as unrelated could be the seed for the generation of creative and somehow surprising artifacts. For example, *time* and *money* could seem unrelated things, but if we consider that both are valuable, then we can establish a metaphor as famous as “*time is money.”*

In order to explore this idea we need to rely on resources based on concepts and their relations, so we can explore and establish associations between unseemingly related ideas. As we saw in section 2.1, structured resources such as WordNet employ vertical thinking to obtain conceptual similarity. This type of thinking only returns the most similar concepts, but our goal is to find similar and not-so-obvious concepts, so we need to use lateral thinking. In consequence, we need a conceptual structure where every concept is placed not only taking into account its conventional usage but its diverse and unconventional usages (Veale, 2014b). For this purpose, we have made use of Thesaurus Rex (Veale and Li, 2013), since it is a resource that provides lateral views of concepts. Thesaurus Rex is explained in detail in section 3.1 and the process applied to finding concept associations using this resource is presented in section 3.2. The concept-concept associations obtained by our approach are not useful for themselves, so we have explored their use for creating two different kinds of creative artifacts (rhetorical figures and riddles) as explained in section 3.3. In addition, by using Thesaurus Rex instead of other, more structured resources, we intend to build upon the Neural Theory of Language, as the knowledge used to find the concept associations is mined from everyday language (influenced by people's embodied experiences), instead of more orthodox resources such as dictionaries (which provide more objective sources of knowledge and tend to set subjective considerations aside).

### 3.1. Thesaurus rex

Thesaurus Rex organizes concepts according to the categories into which they are placed by speakers in everyday language (*food, drink, animal*…). These categories have an associated weight that represents their relative salience for the given concept. Thesaurus Rex can show different categories for each concept and allows in turn to consult other concepts in each category. For example, some of the most salient categories for the concept *cookie* are *food* or *item* and some of the least salient are *code* or *user*. Concepts in Thesaurus Rex have also properties or modifiers which are accompanied by a weight indicating how strong its relation to the concept is. For example, for *cookie* some of the most salient modifiers are *sweet, baked* or *sugary*, and some of the least salient are *extra* or *portable*. It is important to note that Thesaurus Rex does not distinguish different meanings in polysemic words, as in the case of *cookie* (the sweet cookie vs. the computer cookie). However, the weight of categories and modifiers may be influenced by the usage of a certain meaning.

**Figure 1:**
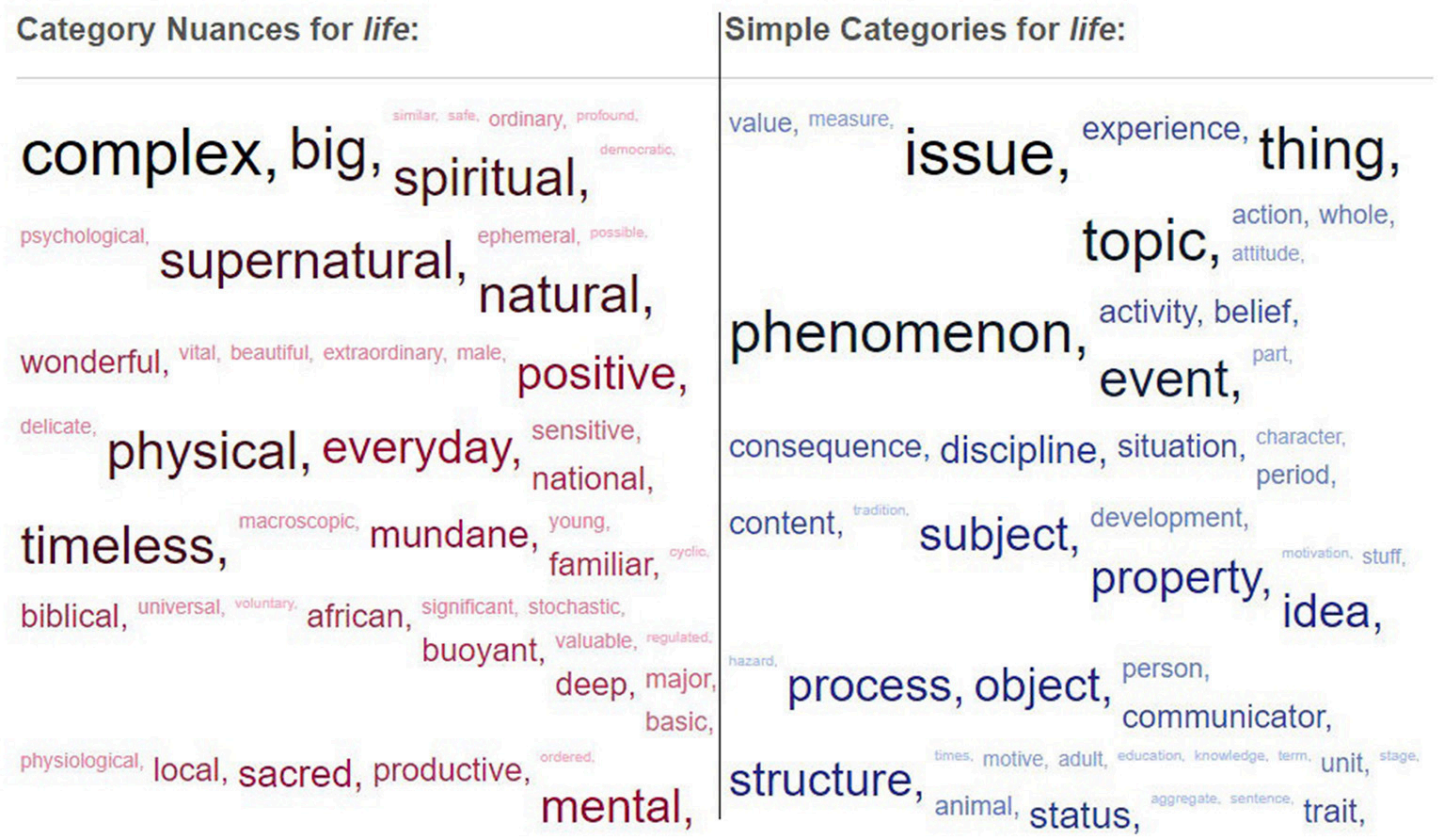
Screenshot from the Thesaurus Rex web application for the word *life*, showing the information about the modifiers (*category nuances*) that can be applied to it, and the categories (*simple categories*) it belongs to.

Thesaurus Rex can be consulted and explored through a web application^3^. For example, in Figure 1 we can see the information obtained for the word *life*, with *issue* and *phenomenon* as some of the categories *life* belongs to, and *complex* or *big* as some of its possible modifiers. The weight representing the salience of the relation between the concept and its category or modifier is displayed using the size of the text, with bigger texts corresponding to higher weights. Thesaurus Rex can also be used in external applications as a web service, and it is possible to download all the information about categories, modifiers and weights through an XML file.

### 3.2. Using thesaurus rex to find concept associations

The proposed approach receives a common noun as an input corresponding to the target concept for the association. The system then looks for related concepts associated with the target concept through properties and categories. The output is a pair concept-property where the newly discovered concept is somehow related to the original one through the obtained property. The returned property is shared between both concepts and is a salient property for both. For example, if *life* is received as an input in our system, one possible output will be *politics* as the associated concept and *complex* as the shared property between *life* and *politics*. The detailed process is described below, along with an example using *life* as a target to illustrate the process:

1. **Selection of target concept categories**. In order to obtain the categories to which the target concept belongs, first we extract all the categories of the target concept using a Thesaurus Rex query. From this list, only the *N*% of the categories with the highest weights are considered as candidates. The value of *N* is configurable. If a high *N* value is set, we will obtain a list of categories with lower weights, and therefore less relevant to the target concept. Similarly, we can set *N* to a low value, facing the risk of shortening the list to a single element. In the case of *life*, some of the categories with the highest weights in Thesaurus Rex are *issue* and *phenomenon*, as shown in the *Simple Categories* part of Figure 1.
2. **Selection of target concept modifiers**. In addition to the categories we also need a list of modifiers associated to the target concept, which is returned by a new query to Thesaurus Rex. From this list, the *N*% of attributes with the highest weights are considered as candidates. For example, for the target concept *life*, some of the most salient properties are *natural, big, complex, physical* and *spiritual*, as shown in the *Category Nuances* part of Figure 1. Then, one property out of this list of modifiers is randomly selected. This random selection makes the system less repetitive and allows the exploration of more unusually related concepts, as the associations to the same target concept will not always be the same as if only the modifier with the highest weight was selected. For the current example, the system has chosen the modifier *complex*.
3. **Selection of categories for the chosen modifier**. Using the modifier obtained in the previous step, a new query to Thesaurus Rex is performed in order to obtain categories that present this modifier as a highlighted property. In the *life* example, the categories selected could be *issue, system*, and *organism* which are categories that present the *complex* property in Thesaurus Rex. One of these categories is then selected. The system has two possible configurations at this point: to select a category that contains the target concept (a category that appears in the list obtained in step 1), or a category in which the target concept is not included (discarding categories that match those obtained in step 1). If we choose the first option we will be giving more weight to vertical thinking while if we choose the second option the lateral thinking will be the predominant one. For the current example the first configuration is chosen, so *issue* is the selected category for the *complex* modifier.
4. **Composition of a new modifier-category query**. A new query for Thesaurus Rex is then composed by using the category obtained in the previous step and the modifier selected in step 2. In the current example, this new query is *complex issue*.
5. **Retrieval of final associated concepts**. With the query composed in the previous step, we obtain a list of concepts that belong to the category selected in step 3 (*issue*) and at the same time present the property selected in step 2 (*complex*). This list is usually quite extensive, so the system randomly chooses among the *N*% of concepts with the highest weights. In our example, the final concepts associated to the target concept are *education, politics* and *ethics*. One of these concepts is randomly selected for creating the final concept association.

Table 1 shows a few examples of target concepts and how our approach obtains concepts associated to them. The choices in each step are presented in bold, and the *writer* example has been repeated so the effect of different selections can be appreciated. We can see that some of the associations are more unusual than others. For example, we can consider that *life* is not initially related to *education* or *politics*, whereas *writer* and *artist* (second example) are much more similar. However, if in step 3 the chosen category is *person* instead of *individual* (third example), we can obtain much more interesting associations.

**Table 1:** Examples of obtained concept associations.

	**Step**	***Life***	***Writer***	***Writer***
1	Categories	issue, phenomenon…	worker, individual…	worker, individual …
2	Modifiers	natural, big, **complex**, physical, spiritual…	cultural, **creative**, professional…	cultural, **creative**, professional…
3	Categories for the selected modifier	**issue**, system, organism…	person, **individual**…	**person**, individual…
4	New query	**complex issue**	**creative individual**	**creative person**
5	Obtained concepts	education, politics, ethics…	artist, designer, musician…	Leonardo Da Vinci, Tim Burton, Tony Stark…

*Words in bold represent the choices made in each step of the process. The writer example is repeated so the effect of a different choice in step 3 can be appreciated*.

### 3.3. Generation of rhetorical figures and riddles from the obtained associations

From the point of view of rhetorical figures, we are interested in figurative language expressed in the form of phrases with special meanings not based on the literal meaning of the words. For this work we have explored the generation of three types of rhetorical figures or tropes that have in common the connection between two concepts:

- *Metaphor*: A metaphor is a widely-used literary mechanism which allows a comparison between two divergent concepts. Metaphors transfer the qualities of one word to another, as in the famous metaphor from Shakespeare in “As you like it”: *World is a stage and men are merely players*. Here, the qualities of *stage* and *players* are transferred to *world* and *men*.
- *Simile*: A simile is a kind of metaphor where the comparison is made using the words “as” or “like”, as in *The truth was like a bad taste in his mouth*. In a simile two different concepts are compared to evolve a new meaning. However, the final concept is considered like the original one, but cannot totally be substituted by it.
- *Analogy*: An analogy is a literary mechanism which links two dissimilar concepts by common properties, as in *You were as brave as a lion*. Here, the quality of being *brave* (the property) is used to link *lion* (the similar concept) to *you* (the original concept).

Considering the well-known metaphor *Time is money* as an example, the concept that is talked of rhetorically (*time* in this case) is known as the target and the concept that provides the rhetorical figure (*money*) is known as the source.

We consider these three kinds of rhetorical figures to be suitable for the purpose of this work based on the idea stated by Black (1955) that made explicit that metaphors depend upon conceptual connections between networks of concepts. Inherent to this approach is the idea that metaphors are a matter of cross-domain mapping (Lakoff, 1993). Therefore, a metaphor can be understood as a cognitive process that builds or maps connections between networks of concepts as it occurs also with similes and analogies.

In order to generate these rhetorical figures we follow a template-based method. We make use of the approach described in section 3.2 to find concept associations using the provided target concept of the rhetorical figure as an input to obtain the SOURCE and the PROPERTY which are needed to fill in the gaps of our templates. The simplest and purest form for analogies, similes and metaphors are used to create these templates:

- Analogy: *TARGET is as PROPERTY as SOURCE*.
- Simile: *TARGET is like SOURCE*.
- Metaphor: *TARGET is SOURCE*.

For example, for the target concept *life* (see Table 1) our system returns the following rhetorical figures:

- *Life is as complex as politics*.
- *Life is like politics*.
- *Life is politics*.

For the generation of riddles a template-based method is also used. The basis of the generated riddles is the mapping between a target concept (i.e. *writer*) and other entities. To carry out the mapping, the properties shared by the concepts are taken into account (i.e. *creative*). The resulting riddles are composed as a sequence of comparisons following this template: “What is…as *attribute* as *concept*?”, where *attribute* is a property of the target concept which is the answer to the riddle, and *concept* is a different entity that shares the value of the *attribute* with the target concept.

In order to generate a riddle for a target concept we use the approach presented in section 3.2 using the target concept of our riddle as input. The output obtained (PROPERTY and SOURCE) is then used to fill in the template “as *attribute* as *concept*?” as many times as desired (determined in the system configuration). In each round, a new comparison is generated and added to the final riddle. A possible riddle for the target concept *writer* (see Table 1) with three comparisons could be the following:

What is …
…*as creative as Leonardo Da Vinci?*
…*as cultural as a book?*
…*as professional as a teacher?*

## 4. Evaluating the suitability of concept associations for the generation of rhetorical figures and riddles

In order to assess whether the concept associations obtained by our system are useful from a computational creativity point of view, we evaluated the two kinds of creative artifacts we are generating. Regarding rhetorical figures, our purpose was to determine the quality of the generated metaphors, similes and analogies and how different configurations influence the obtained results. Regarding riddles, we assessed if the obtained concept associations are useful for creating riddles that can be guessed by final users.

### 4.1. Evaluation of rhetorical figures

The purpose of this evaluation was to test whether concept associations obtained by our approach (see section 3.2) are useful for creating natural sounding rhetorical figures, and how using different configurations and types of target concepts influence the obtained results.

#### 4.1.1. Methods

In order to have two different baselines in our experiment to measure the quality of the generated figures, we have used a set of commonly accepted rhetorical figures, along with a set of random figures that were created manually by us. The aim is to compare them against the ones generated by our system and assess if evaluators find great differences between them. In addition, we intended to test the idea presented in Deignan (2005) about how typically the target in rhetorical figures is an abstract concept^4^ and the source a concrete one^5^. In order to do that, we have used both abstract and concrete concepts as inputs to generate the set of rhetorical figures to be evaluated.

The evaluation set was composed by 36 analogies, 36 similes and 36 metaphors. The entire list of rhetorical figures used in the evaluation can be seen in Appendix A at the end of the paper. To create these elements, 36 different words were used as target concepts and one analogy, one simile and one metaphor were created for each of them. In order to avoid the possibility that one evaluator could evaluate several rhetorical figures related to the same target concept, the original data set was divided into three different subsets of 36 rhetorical figures. Each subset had 12 metaphors, 12 similes and 12 analogies, all of them created from a different target concept.

The way in which the analogies, similes and metaphors were created was the following:

- Commonly accepted figures: 6 concepts (3 abstract and 3 concrete) were used as target concepts to obtain commonly accepted metaphors, similes and analogies. These concepts were TIME, KNOWLEDGE, ARGUMENT, BALLERINA, STAR and THUNDER. We looked for commonly used figures of speech such as *Time is money* in both the book “Metaphors We Live by” (Lakoff and Johnson, 1980) and METALUDE^6^, a metaphor database created in Lingnan University.
- Random figures: 6 concepts (3 abstract and 3 concrete) were used as target concepts to obtain random metaphors, similes and analogies manually by us. To generate this subset we randomly selected words from a dictionary to be the source and the property shared by the two concepts of our metaphors, similes and analogies. The target concepts were HUNGER, SAILING, SYLLOGISM, ELEPHANT, CORKSCREW, and TRAIN.
- Automatically generated figures: 24 words (12 abstract and 12 concrete) were used as target concepts by our system to obtain metaphors, similes and analogies. These words were obtained randomly from a list of common words in English. As it was explained in section 3.2, the system could be parametrized to select the source from the same category as the target concept or from a different category. In order to test if this choice could influence the obtained results, half of the automatically generated figures were generated with the system configured to obtain the source concept from the same category as the target, and the other half to take the source concept from a different category. The chosen concepts were:
  - For the same category configuration (6 abstract and 6 concrete): WEDDING, WISH, LIFE, ANGEL, DEVIL, GOVERNMENT, SNOW, NEEDLE, COTTON, HONEY, BATTLE, and WRITER.
  - For the different category configuration (6 abstract and 6 concrete): SAVING, ACCIDENT, NETWORK, IDEA, ASSEMBLY, WINTER, MOON, REFUGEE, TEMPLE, ACID, BULLET, and DRAWER.

The evaluation was carried out as an online survey using Google Forms, in such a way that three questionnaires were created, one for each of the subsets already mentioned above (each subset with 12 metaphors, 12 similes and 12 analogies from different target concepts). Each evaluator received a link to one of the three surveys and was asked to score each of the rhetorical figures using a Likert scale. Evaluators were asked to rate how appropriate or natural sounding each figure was using a score from 1 to 7 (where 1 symbolizes a completely inappropriate figure and 7 represents a completely natural sounding one).

In order to analyze the results, we used the mean along with the typical deviation and the median along with the interquartile range. Data were analyzed by one-way ANOVA, followed by inspection of all differences by Duncan's multiple-range test. Differences were considered significant at *P* < 0.05.

#### 4.1.2. Results and discussion

The evaluation was carried out by 72 evaluators, in such a way that each of the three subsets of rhetorical figures was assessed by 24 different evaluators. The overall evaluation (shown in Table 2) results were as expected: random figures turned out to be the ones with the lowest ratings (mean = 1.77 and median = 1.52) and commonly accepted figures obtained the highest ratings (mean = 5.08 and median = 5.10). It is important to note that not even the commonly accepted rhetorical figures obtain the highest score (7), which indicates that the evaluators were reluctant to assign maximum scores. Regarding automatically generated figures, the general obtained mean is 4 (3.19 for those of different categories and 4.20 for those belonging to the same category) which indicates that although the process we have used to generate the rhetorical figures works quite well when concepts of the same category are used, according to the opinions of the evaluators, something happens in the case of using concepts that belong to different categories, which, in general, obtain worse results. We have applied One-Way Anova with Duncan's multiple range test to test the significance of this conclusions and the result (*P* < 0.000001) confirms its statistical significance. Looking at the results we can conclude that the scores of the tropes generated by our system are closer to the scores given to the figures commonly accepted than to the ones of the random tropes.

**Table 2:** Overall results for the evaluation of rhetorical figures.

**Source**	**Total number of figures**	**Mean**	**Std. dev**.	**Median**	**Quartile range**
**Random**	18	1.77	0.61	1.52	0.88
**Commonly accepted**	18	5.08	0.77	5.10	1.08
*Generated (different category)*	36	3.19	1.13	3.21	2.00
*Generated (same category)*	36	4.20	1.13	4.13	1.60
**Generated (total)**	72	3.70	1.23	3.83	1.73

We also examined the data obtained for each kind of figure: metaphors (Table 3), similes (Table 4) and analogies (Table 5). As we can see, in all cases the random metaphors are rated as meaningless by the evaluators with mean values between 1.5 and 2. In contrast, commonly accepted figures get the highest results, with mean values between 4.9 and 5.3, although proving that evaluators did not always prefer the maximum score. Once again, the results of the generated tropes (mean values between 3.5 and 4) are closer to the results of the commonly accepted figures. To test the statistical significance of the comparison between methods (random, commonly accepted and generated) by resource (metaphor, simile and analogy) we used One-Way Anova with Duncan's test for multiple comparisons. The results (*P* < 0.000001 for the analogies, *P* = 0.001435 for the metaphors and *P* = 0.000076 for the similes) confirm that the comparisons made are statistically significant.

**Table 3:** Metaphor evaluation results.

**Source**	**Number of metaphors**	**Mean**	**Std. dev**.	**Median**	**Quartile range**
**Random**	6	1.72	0.66	1.44	1.13
**Commonly accepted**	6	4.88	0.95	5.04	1.04
*Generated (different category)*	12	2.63	1.07	2.40	1.63
*Generated (same category)*	12	4.28	1.53	4.60	2.58
**Generated (total)**	24	3.46	1.55	3.33	2.58

**Table 4:** Simile evaluation results.

**Source**	**Number of Similes**	**Mean**	**Std. dev**.	**Median**	**Quartile Range**
**Random**	6	2.02	0.77	1.88	1.17
**Commonly accepted**	6	5.10	0.50	4.92	0.75
*Generated (different category)*	12	3.10	1.22	2.88	2.29
*Generated (same category)*	12	4.21	0.89	4.00	1.06
**Generated (total)**	24	3.65	1.19	3.85	1.44

**Table 5:** Analogy evaluation results.

**Source**	**Number of analogies**	**Mean**	**Std. dev**.	**Median**	**Quartile range**
**Random**	6	1.56	1.56	1.48	0.21
**Commonly accepted**	6	5.26	0.89	5.44	1.63
*Generated (different category)*	12	3.83	0.78	4.04	1.15
*Generated (same category)*	12	4.12	0.97	4.10	1.46
**Generated (total)**	24	3.97	0.87	4.04	1.27

Looking at the results of the generated tropes, we can see that ratings for analogies (Table 5) are slightly higher than in the two previous figures (Tables 3, 4), probably due to the fact that the property shared by the two concepts is explicitly stated, so the evaluators can see the reason why the system considers the two concepts related to each other and they are more inclined to accept it as valid. This comparison is not statistically significant but is very close to being based on the results of One-Way Anova with Duncan's test (*P* = 0.541774).

When we continue to analyse the subsets, we can see that the results obtained for the generated figures belonging to different categories are less promising than those obtained for the ones within the same category. That points out that sharing a property but not a category could not be enough to generate a good rhetorical figure.

Regarding the type of target concepts (abstract or concrete) we can see the results in Table 6 grouped by source and in Table 7 grouped by type of rhetorical figure (excluding the random tropes which are not relevant for this analysis and could contaminate the results). It can be seen that evaluators prefer metaphors based on abstract concepts while for analogies they prefer those based on concrete concepts. Regarding the relationship between the type of target concepts and the source of the tropes there is a slight preference for abstract concepts except in the case of figures generated using concepts of the same category. In this case, concrete concepts are preferred. This results are not statistically significant (we have applied T-Student test to prove it) so it will be necessary to carry out more studies to obtain stronger conclusions.

**Table 6:** Results according to the type of concept grouped by source.

**Figure**	**Concept type**	**Number of elements**	**Mean**	**Std. dev**.	**Median**	**Quartile range**
**Commonly accepted**	Abstract	9	5.27	0.65	5.54	1.08
	Concrete	9	4.89	0.88	5.00	0.63
*Generated (different category)*	Abstract	18	3.03	1.11	3.21	1.58
	Concrete	18	3.34	1.16	3.27	2.13
*Generated (same category)*	Abstract	18	4.31	1.21	4.08	1.92
	Concrete	18	4.10	1.07	4.13	1.38
**Generated (total)**	Abstract	36	3.67	1.31	3.79	1.58
	Concrete	36	3.72	1.17	4.04	1.92

*The 9 abstract/concrete commonly accepted figures correspond to 3 metaphors, 3 similes and 3 analogies, and the 18 abstract/concrete generated figures correspond to 6 metaphors, 6 similes and 6 analogies, as explained in section 4.1.1*.

**Table 7:** Results according to the type of concept grouped by rhetorical figure.

**Figure**	**Concept type**	**Number of elements**	**Mean**	**Std. dev**.	**Median**	**Quartile range**
Metaphors	Abstract	15	3.87	1.69	3.83	3.25
	Concrete	15	3.61	1.43	3.63	2.79
Similes	Abstract	15	4.04	1.39	4.00	1.37
	Concrete	15	3.84	1.09	4.08	1.67
Analogies	Abstract	15	4.05	1.04	4.04	1.50
	Concrete	15	4.41	0.98	4.29	1.04

*Number 15 corresponds to 3 commonly accepted figures, 6 generated with different categories and 6 generated with the same category, as explained in Section 4.1.1*.

We can conclude that, although the process we have used to generate the rhetorical figures works quite well when concepts of the same category are used, something happens in the case of using concepts that belong to different categories. This fact points to the need of using additional properties or relationships in order to obtain concepts that can subsequently give rise to more meaningful rhetorical figures.

### 4.2. Evaluation of riddles

We have carried out an evaluation to test whether the concept associations obtained by our system provided useful seeds for riddle generation, while at the same time assessing the quality of the resulting riddles. In order to do that, human evaluators were asked to guess the target concepts which were used to create the riddles. Then, we studied the rate of success obtained by the evaluators, while at the same time we analyzed how many clues (in the form “What is…as *attribute* as *concept*?”) were required to obtain the correct answers in different riddles.

With this evaluation we test if it is possible to guess the riddles generated by our system as it was done in Guerrero et al. (2015) (see section 2.3). We consider this is the first step to evaluate the quality of the generated riddles, since if created riddles are impossible to guess, then they can not be considered as riddles and therefore there would not be anything else to analyze. Once we have tested that our riddles can be solved, our next steps aim at measuring other aspects such as creativity, sound or originality as it was done with JAPE (see section 2.3), but we have postponed this task for future work.

#### 4.2.1. Methods

Ten different riddles were presented to human evaluators to see if they were able to find the initial target concepts. Riddles were presented in four phases, in such a way that we could see how many comparisons were needed to solve the riddle. In the first phase a single clue was presented, in the second phase two clues were presented (the first one and a new one), three clues in the third phase and, finally, four in the fourth phase. In each phase evaluators were asked to give their best guess, which they could reuse or change in subsequent phases. Each evaluator was presented with the four phases of each riddle even if they had guessed it correctly in one of the first three. The evaluation was carried out using a questionnarie in Google Forms and some personal information was collected for statistical purposes (age, gender and riddle ability).

When generating the riddles for the evaluation, we realized that some of the clues (“as …as …”) did not add new information to previous ones, or the information added was contradictory or not valid due to the polysemy of some concepts. For example, this was the case for *coke*, which is a flavored carbonated drink and the street name for cocaine. Our system had generated “*is as carbonated as …*” and “*is as hard as …*,” where the second comparison was obtained due to the word association “*coke-hard drug*.” Examples of contradictory clues are mostly related to attributes with imprecise values, like size or age. For example, Thesaurus Rex categorizes the concept *dog* both as small and as large depending on the context. However, if our system chooses both attributes, we would have contradictory clues in the riddle.

In order to carry out a more detailed evaluation, we decided to create two different riddle sets. Using the same ten concepts, but with some differences in the provided clues, we created an original and a curated version of each riddle. For the first set, the first four clues obtained from the concept association system were used. For the second set, we obtained seven different associations and the most significant four were manually selected in order to avoid invalid clues due to polysemic or semantic contradictions. The entire set of riddles used in the evaluation can be seen in Appendix B at the end of the paper.

#### 4.2.2. Results and discussion

Both the evaluation with the original riddles and the one with the curated versions were performed in parallel by 12 different evaluators each, making a total of 24 participants in the experiment. The order of appearance for each riddle was fixed, so all the evaluators participating in each part of the experiment were presented exactly with the same riddles.

The overall evaluation results are shown in Table 8. This Table shows the mean, the median, the min and the max number of riddles guessed per person in each phase. In each phase the number of riddles was 10, so the maximum possible number of riddles guessed per person is also 10. For the original set of riddles, all the participants made at least two correct guesses (5%) and 13 at most (32.5%), with an average of eight correct guesses (20%) per person. For the curated set, the minimum amount of correct guesses was seven (17.5%) and the maximum was 24 (60%), with an average of 17.5 correct guesses per person (43.75%). It is important to note that the aim was not to get all the riddles right, which would indicate that the generated riddles were too easy. We can conclude that the results of the curated version of the riddles are better than the ones of the original set, which suggests that an additional selection of comparisons is needed in some cases in order to improve the quality of the generated riddles. The differences are significant as shown by the result of the Student's *T*-test (*P* <.0001) that we apply to the data. If we compare these results with those obtained by Guerrero et al. (2015) we can conclude that the average guesses per person in our system (43.75%) are much better than those obtained by the Guerrero system (15.5%).

**Table 8:** Number of guessed riddles per person for each set.

		**Number of riddles**	**Guesses**			
			**Mean**	**Median**	**Min**.	**Max**.
Original Set	Phase 1	10	0.50	0.00	0.00	2.00
	Phase 2	10	1.58	1.00	0.00	3.00
	Phase 3	10	3.17	3.00	1.00	5.00
	Phase 4	10	2.75	3.00	0.00	4.00
	Total	40	8.00	8.50	2.00	13.00
Curated Set	Phase 1	10	1.08	1.00	0.00	4.00
	Phase 2	10	4.58	5.00	2.00	6.00
	Phase 3	10	5.75	6.50	2.00	8.00
	Phase 4	10	6.08	6.50	3.00	9.00
	Total	40	17.50	18.50	7.00	24.00

Regarding the number of clues needed to guess the correct answer, a curious fact can be seen in Table 8. With just a single clue, there is almost no chance of guessing the target concept. In most cases, people answer at random because there are lots of concepts that could correspond to the presented clue. When the riddle contains two clues, users multiply by four the number of correct answers. When the evaluators are given three clues, in the case of the original set, the number of hits reaches its maximum value. However, in the case of the curated set, with three clues the evaluators guessed an average of 5.75 riddles per person, which is almost the maximum number of hits, making the difference with the last phase (where four tracks are provided) almost insignificant. To validate this conclusion we have carried out a one-way repeated measures ANOVA -corrected by the Bonferroni criterion- which can be seen in Table 9. All the comparisons are statistically significant at *P* < 0.05 except for the comparison between Phase3 and Phase4, which confirms that the best results are obtained with three clues.

**Table 9:** One-way repeated measures ANOVA to test the effect of the number of cues in the number of guessed riddles.

	**Phase 1**	**Phase 2**	**Phase 3**	**Phase 4**
Phase 1		0.03182	0.00012	0.00225
Phase 2	0.03182		0.00384	0.06962
Phase 3	0.00012	0.00384		1.00000

To view the results for each riddle, Tables 10, 11 show the disaggregated number of evaluators that guessed each riddle in each phase. The list of riddles used in the evaluation can be found in Appendix B. In order to analyze the results, it is worth remembering that 12 evaluators participated and therefore the greatest possible value by riddle and phase is 12. Looking at the results in the curated set (Table 11), we can see that R10 has, in the best case scenario, a success rate of 8% (only one evaluator guessed the riddle). This is due to the fact that the attributes selected are not specific enough and there is a large amount of common properties with other concepts. In this riddle, where the target concept was *aircraft*, the attributes selected were *mechanical, fast, mobile*, and *complicated*. However, riddle R3 has a success rate, in the fourth phase, of 92% (only one evaluator failed). In this case, the attributes selected for the target concept (*sun*) were much more specific (*stellar, yellow, hot, central*) and limits the possible answers for this riddle.

**Table 10:** Number of evaluators who guessed each riddle in the original set.

**Phases**	**R1**	**R2**	**R3**	**R4**	**R5**	**R6**	**R7**	**R8**	**R9**	**R10**
Phase 1	2	0	2	1	0	0	0	1	0	0
Phase 2	1	0	8	0	4	3	1	2	0	0
Phase 3	1	0	7	4	6	11	1	8	0	0
Phase 4	1	0	5	2	6	8	0	9	2	0

**Table 11:** Number of evaluators who guessed each riddle in the curated set.

**Phases**	**R1**	**R2**	**R3**	**R4**	**R5**	**R6**	**R7**	**R8**	**R9**	**R10**
Phase 1	0	3	0	2	0	1	2	0	5	0
Phase 2	4	6	9	4	3	7	8	9	5	0
Phase 3	5	5	9	5	7	10	9	9	9	1
Phase 4	5	6	11	6	7	10	9	9	9	1

Looking at the results of the original set (Table 10) we can see that the number of correct answers decreases slightly in some cases (R1, R3, R4, R6, and R7). The reason for this, as explained by the participants in the evaluation, is that sometimes the last clues were contradictory and users were confused and ended up changing their answer in the last attempt. For example, in the case of R6, the target concept was *diamond* and the attributes selected were *transparent, pure, expensive* and *simple*. The last clue (*simple*) contradicts the third one a little (*expensive*).

## 5. Conclusions and future work

The work presented in this paper has the aim of exploring how concept associations can be used to produce creative artifacts such as rhetorical figures and riddles. When two concepts that seem initially not related are linked, we can find associations as creative as *writer-creative-Tony Stark* or *snow-soft-carpet*. The presented evaluation points out that the concept associations created by our system are useful for generating rhetorical figures and riddles of a reasonable quality. However, the evaluation also shows that some confusing associations may be generated when the target of the generation is a polysemic concept or presents some contradictory attributes. Thesaurus Rex does not include any information about different senses of a searched concept, and all the categories and modifiers are mixed without taking polysemy into account. Therefore, it is necessary to develop some mechanisms external to Thesaurus Rex to select only the modifiers related to the sought meaning of the target concept, and consider in a special way attributes with imprecise values that depend on the context, such as *big* or *small* (for example, a dog is big compared with an ant but small compared with an elephant).

However, the use of Thesaurus Rex has set the basis for further work with neuroscientists, cognitive linguists and experimental psychologists, as the process of concept mapping that has been used to generate both rhetorical figures and riddles takes implicitly into account the embodied experiences provided by the nature of the knowledge mined to build Thesaurus Rex. Even though this knowledge presents inconsistencies due to the very nature of human language and mind, the possibility to automatically generate linguistic artifacts (such as conceptual metaphors) that take embodied knowledge into account can constitute a highly valuable tool that allows cognitive scientists to study how our brain works when creating and understanding these metaphors, and to furtherly expand the neural theory of language.

From the point of view of the generation of rhetorical figures, one of the clearest conclusions is that our system generates figures of significantly higher quality when considering concepts in the same category than when considering different categories. However, some of the best rhetorical figures in real life are constructed with concepts from different categories, such *time is money* or *love is a journey*, corroborating that creativity needs to combine vertical thinking with lateral thinking as explained in section 2.1. Therefore, we have to explore new approaches when generating figures using concepts in different categories so we can obtain better figures in this case. For example, our approach may be improved relating the original concept with concepts that have more than one property in common. For example, in *a ballerina is a swan*, both concepts share several properties such as *pretty, graceful* and *stylized*. With respect to the amount of information provided in the rhetorical figure, the best results are obtained for analogies. This is probably due to how the property belonging to the source and target concepts is made explicit, and therefore the association between concepts can be more easily understood.

The results obtained indicate that further attempts should be made to evolve our system and generate higher quality rhetorical figures, progressively improving the quality of the system results toward those of rhetorical figures generated by people. The analysis of figurative language databases could be useful in order to find features and traits that are present in commonly used rhetorical figures. For example, we can explore these databases in order to check the degree of similarity between the source and target concepts of a given association. Then, if the concepts are too similar like in “*a writer is like an artist”* the resulting rhetorical figure can be considered correct but not very practical or creative.

Additionally, we must take into account that trying to match the results obtained by commonly accepted figures is probably not possible. Gentner and Bowdle (2001) and Bowdle and Gentner (2005) propose the Career of Metaphor Model, which suggests that conventional and novel metaphors are processed differently. Although both kinds of metaphors should be somewhat cognitively taxing due to an initial stage for structural alignment that is needed for mapping two different concepts, novel metaphors should be more difficult due to always having to compare concepts and generate these mappings on the fly.

From the point of view of riddle generation, the evaluation results suggest that the order in which the comparisons are provided is relevant in order to solve the riddle using less comparisons, so it may be useful to analyze the discriminating power of each attribute, so that the complexity of the riddles can be controlled. If this information is available, the system could select first (or last) the most discriminating attributes of the concept automatically. The underlying idea is that the higher the discriminating power of the attribute is, the easier the riddle will be, as more concepts are excluded from the possible answers. Depending on the desired difficulty of the riddle, we can play with the order of the attributes according to their discriminating power.

In future work, we will explore the generation of more creative riddles, adding for example rhymes as in the example from *The Hobbit* presented in the introduction. This could be possible if the choice of source concepts is also guided by their pronunciation. In addition, we would like to include human created riddles in future evaluations, so we can assess the naturalness of our riddles in comparison with human-made ones. Once we have these improvements incorporated in our riddle generator we will have to evaluate not only if the riddles can be solved, but also creativity, originality or sound of the generated riddles.

Finally, we would like to explore the applicability of the described techniques in the field of accessibility. Other authors have already reported on the use of riddles to allow children with communication difficulties to develop their linguistic skills (Manurung et al., 2008). Following this idea, we aim at exploring the way in which riddles can be incorporated in the life of people with communication disabilities, or how analogies and similes can be used to explain unknown concepts to people who are learning new vocabulary.

## Ethics statement

The present study was carried out in accordance with the recommendations of national and international ethics guidelines, Código Deontológico del Psicólogo and American Psychological Association. The study did not present any invasive procedure, and it did not carry any risk to the participants' mental or physical health, thus not requiring ethics approval according to the Spanish law BOE 14/2007. All subjects participated voluntarily and gave written informed consent in accordance with the Declaration of Helsinki. They were free to leave the experiment at any time.

## Author contributions

The work presented in this paper is the result of PG's Master's Thesis. The rest of the authors were the Master Thesis' advisors and guided PG throughout her investigation. VF has been in charge of the writing of this paper, coordinating the efforts of all the authors.

### Conflict of interest statement

The authors declare that the research was conducted in the absence of any commercial or financial relationships that could be construed as a potential conflict of interest.

## Footnotes

^1^http://boundinanutshell.com/therex3

^2^Symbols + and *-* are used to indicate if a positive or negative spin is intended.

^3^http://ngrams.ucd.ie/therex3/

^4^Abstract concepts are concepts that does not exist at any particular time or place and cannot be felt or experienced using just the five senses (Wiemer-Hastings and Xu, 2005).

^5^Concrete concepts represent physical entities, defined by spatial boundaries and perceivable attributes and can usually be experienced using one's five senses (Wiemer-Hastings and Xu, 2005).

^6^http://www.ln.edu.hk/lle/cwd/project01/web/home.html

## Appendix A: rhetorical figures used in the evaluation

The complete list of rhetorical figures used in the evaluation is presented in this appendix. For each concept, the figures are presented in this order: metaphor/simile/analogy.

- Commonly accepted figures:
  - TIME: Time is money / Time is like money / Time is as valuable as money
  - KNOWLEDGE: Knowledge is light / Knowledge is like light / Knowledge is as attractive as light
  - ARGUMENT: An argument is a war / An argument is like a war / An argument is as violent as a war
  - BALLERINA: A ballerina is a swan / A ballerina is like a swan / A ballerina is as graceful as a swan
  - STAR: A star is a diamond / A star is like a diamond / A star is as bright as a diamond
  - THUNDER: A thunder is a lion / A thunder is like a lion / A thunder is as mighty as a lion

- Random figures:
  - HUNGER: Hunger is knowledge / Hunger is like knowledge / Hunger is as mechanical as knowledge
  - SAILING: Sailing is boyhood / Sailing is like boyhood / Sailing is as allergenic as boyhood
  - SYLLOGISM: A syllogism is a nation / A syllogism is like a nation / A syllogism is as ungulate as a nation
  - ELEPHANT: An elephant is a napkin / An elephant is like a napkin / An elephant is as holy as a napkin
  - CORKSCREW: A corkscrew is a stamp / A corkscrew is like a stamp / A corkscrew is as furry as a stamp
  - TRAIN: A train is a violin / A train is like a violin / A train is as observational as a violin

- Automatically generated figures:
  - Source and target from the same category:
    - WEDDING: A wedding is a party / A wedding is like a party / A wedding is as private as a party
    - WISH: A wish is a desire / A wish is like a desire / A wish is as mental as a desire
    - LIFE: Life is politics / Life is like politics / Life is as complex as politics
    - ANGEL: An angel is a fairy / An angel is like a fairy / An angel is as invisible as a fairy
    - DEVIL: Devil is love / Devil is like love / Devil is as spiritual as love
    - GOVERNMENT: Government is family / Government is like family / Government is as social as family
    - SNOW: Snow is a carpet / Snow is like a carpet / Snow is as soft as a carpet
    - NEEDLE: A needle is a knife / A needle is like a knife / A needle is as sharp as a knife
    - COTTON: Cotton is cashmere / Cotton is like cashmere / Cotton is as natural as cashmere
    - HONEY: Honey is sugar / Honey is like sugar / Honey is as sticky as sugar
    - BATTLE: A battle is a war / A battle is like a war / A battle is as historical as a war
    - WRITER: A writer is a designer / A writer is like a designer / A writer is as creative as a designer

- Source and target from different categories:
  - SAVING: Saving is farming / Saving is like farming / Saving is as productive as farming
  - ACCIDENT: An accident is an electric shock / An accident is like an electric shock / An accident is as unexpected as an electric shock
  - NETWORK: Network is family / Network is like family / Network is as social as family
  - IDEA: Idea is colors / Idea is like colors / Idea is as abstract as colors
  - ASSEMBLY: An assembly is an aircraft / An assembly is like an aircraft / An assembly is as complex as an aircraft
  - WINTER: Winter is salad / Winter is like salad / Winter is as cold as salad
  - MOON: The moon is an halogen lamp / The moon is like an halogen lamp / The moon is as bright as an halogen lamp
  - REFUGEE: A refugee is an elderly / A refugee is like an elderly / A refugee is as vulnerable as an elderly
  - TEMPLE: A temple is a school / A temple is like a school / A temple is as public as a school
  - ACID: Acid is a tiger / Acid is like a tiger / Acid is as dangerous as a tiger
  - BULLET: A bullet is a bolt / A bullet is like a bolt / A bullet is as metal as a bolt
  - DRAWER: A drawer is a chestnut / A drawer is like a chestnut / A drawer is as dark as a chestnut

## Appendix B: riddles used in the evaluation

The complete list of riddles used in the evaluation is presented in Table A1. The first column, *Riddle*, shows the position of the riddle in the evaluation, and refers to the riddles shown in Tables 10, 11. The second column, *Word*, shows the target concept that had to be guessed by the evaluators. In the third column, *Original riddle*, we show the sequence of clues, that were given to users to guess the target word in the original version of the evaluation. Finally, in the last column, *Curated riddle*, we show the sequence of clues that were provided in the curated version of the evaluation.

**Table A1:** Riddles used in the evaluation.

**Riddle**	**Word**	**Original riddle**	**Curated riddle**
R1	*Ant*	…*as tiny as an isopod?*	…*as invertebrate as a crab?*
		…*as terrestrial as a bird?*	…*as social as a wedding?*
		…*as small as a rabbit?*	…*as tiny as an isopod?*
		…*as social as a wedding?*	…*as annoying as a slug?*
R2	*Devil*	…*as useless as sand?*	…*as evil as envy?*
		…*as common as chromium?*	…*as supernatural as a deity?*
		…*as evil as envy?*	…*as powerful as extreme anger?*
		…*as invisible as joy?*	…*as bad as a war?*
R3	*Sun*	…*as stellar as a galactic nucleus?*	…*as hot as a soup?*
		…*as hot as a soup?*	…*as stellar as a galactic nucleus?*
		…*as natural as wood?*	…*as yellow as a mango?*
		…*as gravitational as a planet?*	…*as central as a living-room?*
R4	*Car*	…*as large as a horse?*	…*as mechanical as a gear?*
		…*as physical as a hardness?*	…*as everyday as clothing?*
		…*as private as a hotel?*	…*as heavy as lead?*
		…*as technical as a medicine?*	…*as large as a horse?*
R5	*Whale*	…*as marine as a barnacle?*	…*as large as a furniture?*
		…*as large as a furniture?*	…*as migratory as a goose?*
		…*as migratory as a goose?*	…*as marine as a barnacle?*
		…*as aquatic as a fish?*	…*as aquatic as a fish?*
R6	*Diamond*	…*as transparent as a hair?*	…*as hard as concrete?*
		…*as pure as gold?*	…*as transparent as a hair?*
		…*as costly as a car?*	…*as precious as silver?*
		…*as simple as a screwdriver?*	…*as geometric as a circle?*
R7	*Milk*	…*as liquid as methanol?*	…*as white as a pollock?*
		…*as raw as cotton?*	…*as liquid as methanol?*
		…*as natural as wood?*	…*as natural as wood?*
		…*as everyday as clothing?*	…*as raw as cotton?*
R8	*Shark*	…*as large as a horse?*	…*as dangerous as scissors?*
		…*as dangerous as scissors?*	…*as marine as a barnacle?*
		…*as marine as a barnacle?*	…*as predatory as a cheetah?*
		…*as aquatic as a fish?*	…*as big as a tiger?*
R9	*Coke*	…*as commercial as a supermarket?*	…*as carbonated as a cooler?*
		…*as hard as concrete?*	…*as commercial as a supermarket?*
		…*as carbonated as a cooler?*	…*as dark as a fig?*
		…*as cool as damp soil?*	…*as cool as damp soil?*
R10	*Aircraft*	…*as physical as swimming?*	…*as mechanical as a pump?*
		…*as mobile as a truck?*	…*as fast as a hamburger?*
		…*as modern as a restaurant?*	…*as mobile as a truck?*
		…*as fixed as a tree?*	…*as complicated as a ship?*
